# Techno-Functional, Antioxidant, and Sorption Properties of Dietary Fiber Concentrates from Guamuchil (*Pithecellobium dulce*)

**DOI:** 10.3390/foods14244316

**Published:** 2025-12-15

**Authors:** Mayra Deyanira Ramírez-Aguirre, Fátima Alfonso-Acosta, Ricardo de Jesús Montiel-López, Tomás García-Cayuela, Viridiana Tejada-Ortigoza, Luis Eduardo Garcia-Amezquita

**Affiliations:** 1 Tecnologico de Monterrey, Escuela de Ingenieria y Ciencias, Ave. General Ramón Corona 2514, Zapopan 45138, Jalisco, Mexico; a01114369@tec.mx (M.D.R.-A.);; 2 Tecnologico de Monterrey, Escuela de Ingenieria y Ciencias, Ave. Eugenio Garza Sada 2501, Monterrey 64700, Nuevo León, Mexico

**Keywords:** legume, dietary fiber, proximate characterization, functional properties, moisture sorption isotherms

## Abstract

Guamuchil (*Pithecellobium dulce*) is an underutilized legume with significant potential as a food ingredient. This study valorized guamuchil fractions (pulp, peel, and seed) by developing dietary fiber concentrates (DFC) and evaluating their physicochemical, techno-functional, and antioxidant properties using a single-factor completely randomized design. Proximate composition and dietary fiber profiles (2011.25) were analyzed following AOAC official methodologies. Results showed the peel fraction contained the highest total dietary fiber (64.16 ± 1.23 g 100 g^−1^ dry basis (db)) and total phenolic content (15.46 ± 0.26 mg GAE g^−1^ db), positioning it as a bioactive fiber source. Conversely, the pulp DFC exhibited superior hydration properties, with high solubility (43.54 ± 1.22%), swelling (10.23 ± 0.30 mL g^−1^ db), and water retention capacity (14.17 ± 0.35 mL g^−1^ db), making it suitable as a texturizer. Moisture sorption isotherms exhibited type II sigmoidal behavior, accurately fitted by GAB and Peleg models (R^2^ ≥ 0.997). The pulp showed higher hygroscopicity and water binding ability, whereas peel and seed fractions displayed lower sorption and enhanced stability. These findings demonstrate that Guamuchil DFCs are suitable as a potential food formulation ingredient owing to their high functionality.

## 1. Introduction

Guamuchil (*Pithecellobium dulce*), a legume that grows wild on trees in the northwestern region of Mexico, is rarely cultivated for consumption due to the lack of adequate industrial information about its nutritional value. Therefore, much of its natural production and nutritional potential gets wasted [1]. Nevertheless, guamuchil has several conventional uses, such as in the preparation of medicine, food, animal feed, and timber. It is also known that indigenous communities in Mexico, such as the Nahua community of Zacualpan, Colima, have a long-standing tradition of utilizing the guamuchil fruit for nourishment and foraging seeds, representing a significant economic impact on the community [2,3].

Guamuchil tree is a medium-sized evergreen tree that belongs to the Fabaceae family and is characterized by a broad, spreading crown that creates a wide canopy with an average height of 5–18 m [4]; it is cultivated during the spring season. *P. dulce* can be found in certain regions of the United States of America, India, Bangladesh, and the Philippines with thin and low, uneven branches, and small dark stems [5]. The fruit peel is linear or curved, measuring up to 20 cm long and 10–15 mm wide; the pods of this fruit hold a red and white spongy pulp, called aril, which covers small black seeds [4,6].

The aril or pulp from the pod is known for its nutritional and medicinal properties. Several studies have revealed the presence of several bioactive phytocomponents in it such as naringenin, quercetin, rutin, gallic acid, clonazepam, and several amino acids such as valine, lysine, phenylalanine, and tryptophan [7,8]. In contrast, recent scientific efforts have focused heavily on the valorization of the seeds, particularly regarding the extraction and functional modification of their protein isolates using emerging technologies [9,10]. However, while the seed protein has been extensively characterized, the dietary fiber potential of the pod’s fractions—specifically the peel and pulp—remains largely underexplored for human consumption. Both fractions contain high amounts of bioactive compounds that can be used to prevent malnutrition [11,12].

Although some recent studies have investigated *P. dulce* peel fibers, the focus has predominantly been on their application as reinforcement in bioactivity (e.g., antimicrobial, antifungal, antioxidant, anti-inflammatory, and anticancer activities) for material sciences rather than as functional food ingredients [13,14,15]. Consequently, the peel covering the pod has yet to be thoroughly evaluated for its physicochemical properties and techno-functional potential in food systems.

In recent decades, dietary fiber has garnered significant attention, not only for its great contribution to health but also for the critical relationship between fiber composition and the functional characteristics of food ingredients [16,17]. The obtention of dietary fiber used to be related to cereals; however, in recent years, other sources, such as fruit, vegetables, and their byproducts, have been explored to extract products rich in dietary fiber, known as dietary fiber concentrates (DFC). In the food processing and formulation industry, the most important characteristics sought in ingredients are their water- and oil-retention capacity, solubility, and the capacity of swelling, emulsifying, and gelation properties [18,19,20,21]. Additionally, for fiber-rich materials, sorption behavior is particularly important because it reflects the distribution of hydrophilic and hydrophobic components, the accessibility of sorption sites, and the potential of these ingredients to act as moisture regulators in complex food systems [22].

Based on the heterogeneous composition of the pod, we hypothesized that the different fractions of guamuchil (pulp, peel, and seed) will yield DFCs with distinct physicochemical and functional profiles, driven by their specific fiber composition and water sorption behavior. Therefore, the present specific objectives of this study were: (1) to conduct a physicochemical characterization of the guamuchil fractions; (2) to obtain DFCs from the pulp, peel, and seed; and (3) to characterize these concentrates in terms of their dietary fiber composition, techno-functional properties, phenolic compounds and antioxidant capacity, and moisture sorption isotherms to determine their suitability as novel ingredients for the food industry.

## 2. Materials and Methods

### 2.1. Reagent and Sample Preparation

General reagents for physicochemical and colorimetric analyses, including DPPH and ABTS, were obtained from El Crisol (Guadalajara, Jalisco, Mexico), while solvents such as ethanol and acetone were supplied by CTR Scientific (Guadalajara, Jalisco, Mexico). For the dietary fiber analysis (AOAC 2011.25), the integrated enzymatic kit and ion-exchange resins (Ambersep 200^®^ H+ and Amberlite FPA53^®^ OH−) were acquired from Megazyme (Wicklow, Ireland).

**Figure 1:**
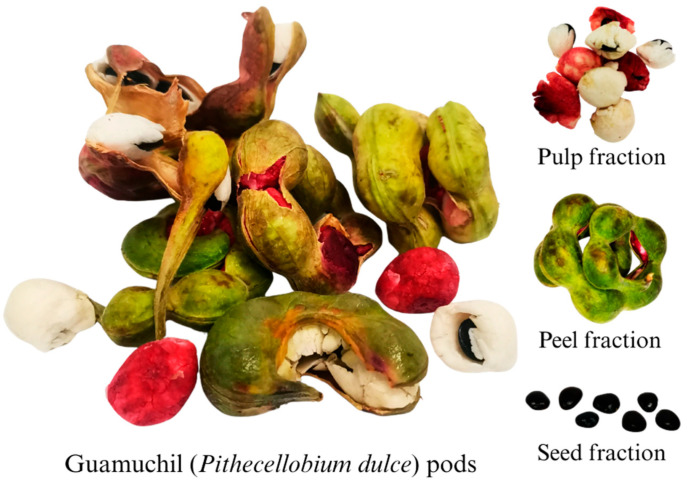
Guamuchil (*Pithecellobium dulce*) pods and fractions.

The raw, ripe guamuchil pods batch were purchased from a local market “Mercado Alcalde” in Guadalajara, Jalisco, Mexico. The pods were cleaned by removing any organic matter and then immersing it in a 1% sodium hypochlorite solution and distilled water for 5 min, followed by rinsing with distilled water. The guamuchil pods (Figure 1) were then categorized into three major fractions: pulp (GuP), peel (GuPe), and seed (GuS).

### 2.2. Physicochemical Composition of Guamuchil Pod

#### 2.2.1. Physical Dimensions and Moisture Content

A conventional vernier was used for the measurement of the length (mm) and diameter (mm) of pods. Mass (g) was measured by using an analytical balance. The moisture content was evaluated as per the AOAC official methodology reported by Garcia-Amezquita et al. [23]. Moisture content was expressed as g 100 g^−1^ of wet basis (wb).

#### 2.2.2. Titratable Acidity (TA) and pH

The AOAC method 942.15 was used to determine the TA, which was expressed as g of citric acid per 100 g of solids. pH was measured by using a potentiometer (Orion Star A211, Thermo Scientific, Waltham, MA, USA).

#### 2.2.3. Soluble Solids (Brix)

The AOAC method 932.12 was used to determine the soluble solids (SS), which were expressed as Brix.

#### 2.2.4. Maturity Index

The maturity index was obtained by using the SS:TA ratio, as proposed by Garcia-Amezquita et al. [23].

### 2.3. DFC Preparation

**Scheme 1:**
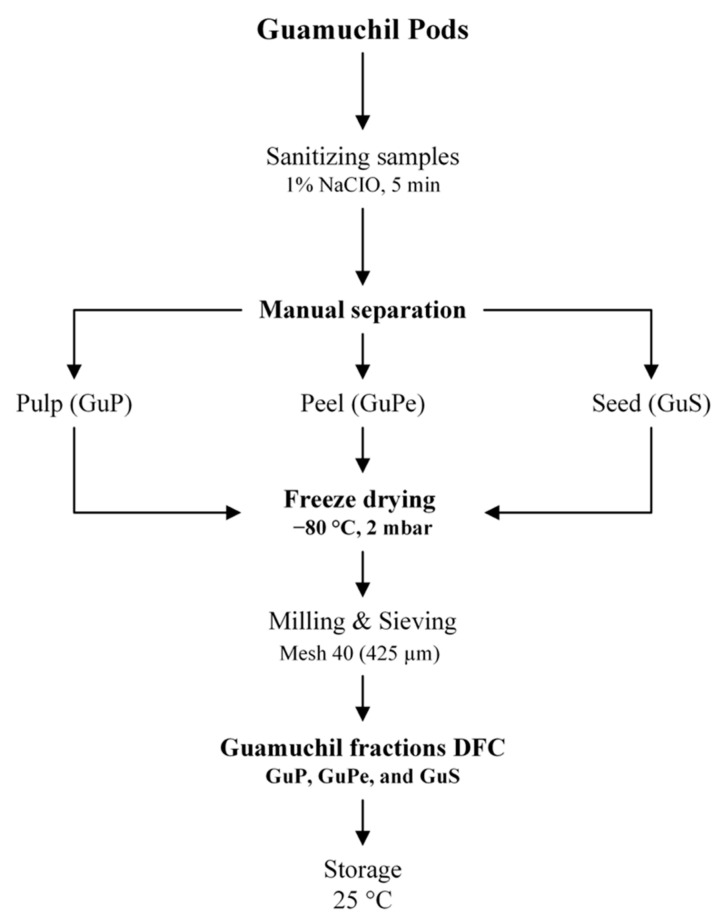
Preparation of Dietary Fiber Concentrates (DFC) from Guamuchil pulp (GuP), peel (GuPe), and Seed (GuS).

The guamuchil fractions were frozen at −80 °C and then freeze-dried using a Labconco Triad (Kansas City, MO, USA), milled (Oster BLSTVB-TV0-013, Atlant, GA, USA), refined (IKA A10 basic, Staufen, Baden-Württemberg, Germany), and sifted (mesh 40). The obtained DFC were stored at 25 °C and protected from direct light for use in further analysis (Scheme 1).

### 2.4. Physicochemical and Proximate Characterization

#### 2.4.1. Dietary Fiber Composition

The dietary fiber composition was evaluated as per the official AOAC methodology 2011.25, albeit with minor modifications, as proposed by Garcia-Amezquita et al. [16]. Total dietary fiber (TDF) is composed of insoluble and soluble dietary fiber (IDT and SDF, respectively). Where SDF is the sum of high (SDFP) and low (SDFS) molecular weight soluble dietary fiber.

#### 2.4.2. Proximate Characterization

The proximate composition of each pod fraction was determined by using the AOAC official methodologies proposed by Garcia-Amezquita et al. [23] for protein (N × 6.25), fat, and ash (methods 920.152, 960.39, and 940.26, respectively). Moisture content was determined by following the same methodology described in Section 2.2.1. The digestible carbohydrate content was calculated by difference. Proximate values were expressed as g 100 g^−1^ of dry basis (db).

#### 2.4.3. Water Activity (a_w_) and Color

The a_w_ was assessed by using an AQUA LAB series 4 TE (Start Decagon Devices, Pullman, WA, USA) at 25 °C. Color was evaluated in the CIELab space with a photocolorimeter CR-410 (Konica Minolta, Tokyo, Japan).

### 2.5. DFC Functional Properties

#### 2.5.1. Techno-Functional Characterization

The techno-functional characterization of the DFCs was evaluated following the protocols previously described by Ramírez-Aguirre et al. [24]. These properties are critical for determining the potential application of fiber ingredients in food systems, influencing texture, stability, and processing efficiency.

Tapping density (TD) provides insight into the packing characteristics, flowability, and compressibility of the powder, which are critical factors for handling, storage, and transportation efficiency. TD was measured by placing a known mass of DFC ($W_{S}$, dry basis) into a 10 mL graduated cylinder. The cylinder was manually tapped on a flat surface for 5 min to settle the powder. The final volume occupied by the sample after tapping $V_{T}$ was recorded. TD was calculated using Equation (1) and expressed as mg mL^−1^.

(1) $$\mathrm{TD}=\frac{W_{S}}{V_{T}}$$

Swelling capacity (SWC) measures the ability of the fiber matrix to expand physically upon water absorption, a property relevant for satiety induction and texture modification in high-moisture food products. To determine this parameter, 200 mg of every DFC were hydrated with 10 mL of distilled water in a large graduated test tube. The suspension was stirred manually with a glass rod to eliminate clumps and ensure proper dispersion. After a hydration period of 18 h at 25 °C, the volume occupied by the sedimented sample ($V_{P}$) was measured. SWC was expressed as mL mg^−1^ using Equation (2):

(2) $$\mathrm{SWC}\;=\frac{V_{P}}{W_{S}}$$

Oil Retention Capacity (ORC) is essential for flavor retention, improving mouthfeel, and stabilizing high-fat food formulations or emulsions. For this analysis, a mixture containing 0.5 g of DFC powder and 10 mL of soybean oil was vortexed for 5 min and subsequently incubated for 18 h at room temperature. The mixture was then centrifuged (3125× *g*, 30 min, 22 °C) (Gyrozen 1580 R, Gimpo, Suwon, Republic of Korea). The volume of the supernatant oil ($V_{S}$) was measured to determine the amount of oil retained by the DFC. ORC was calculated according to Equation (3) and expressed as mL g^−1^.

(3) $$\mathrm{ORC}=\frac{10-V_{S}}{W_{S}}$$

Water Retention Capacity (WRC) and Solubility (SOL) were assessed simultaneously by dispersing 0.5 g of DFC powder in 10 mL of distilled water. The mixture was homogenized using a vortex for 5 min and allowed to equilibrate for 18 h at room temperature. Following this, the suspension was centrifuged (3125× *g*, 30 min, 22 °C). The supernatant was carefully decanted and filtered through a pre-weighed Whatman No. 41 filter paper. Both the pellet (sediment) and the filter paper containing soluble solids were dried at 60 °C for 24 h (DFG-9075A, Qingdao Aucma Global Medical Co., Ltd., Qingdao, China). The weights were recorded before and after drying. WRC (mL g^−1^, dry basis) and SOL (% w w^−1^) were calculated using Equations (4) and (5), respectively:

(4) $$\mathrm{WRC}\;=\frac{10-\;V_{R}}{W_{DP}}$$

(5) $$\mathrm{SOL}=\left(\frac{W_{S}-W_{DP}}{W_{S}}\right)\times 100$$

where $V_{R}$ is the volume of retained water (mL), $W_{DP}$ is the weight of the dried pellet (g), and $W_{S}$ is the initial weight of the DFC powder (g) on a dry basis.

#### 2.5.2. Phenolic Compounds and Antioxidant Capacity

***Methanolic extracts***. Briefly, 0.250 g of the DFC powders were mixed with 15 mL of absolute methanol. The suspension was centrifuged (3125× *g*, 10 min, 4 °C) and the supernatant was transferred into a graduated tube. The pellet was then washed with 10 mL of absolute methanol and re-centrifuged under the same conditions. Subsequently, both the supernatants were mixed and filtered with a Durapore^®^ 0.22-μm PVDF membrane. Finally, the extracts were stored for a maximum of 24 h at 4 °C [25].

***Total phenolic content (TPC)***. For the determination of TPC, the method used by Sánchez-Rangel et al. [26] was followed, albeit with some modifications. Briefly, 60 μL of the methanolic extract was mixed with 960 μL of distilled water and 60 μL of Folin–Ciocalteu’s reagent in a 2 mL tube. The mixture was stirred with a vortex and incubated for 3 min and then combined with 120 μL of a solution of 1 N sodium carbonate. Then, it was incubated for another 30 min, pipetted into a 96-well microplate, and read at 765 nm (Varioskan^TM^ Lux multimode microplate reader, Thermo Scientific, Waltham, MA, USA). The results were reported as mg of gallic acid equivalent (GAE) per g db.

***Antioxidant activity by DPPH and ABTS assays***. For the formulation of the solution of DPPH (2,2-diphenyl-1-picryl-hydrazyl-hydrate), 2.5 mg of the reagent was weighed and added to 100 mL of absolute methanol. Then, 20 μL of the methanolic extract was added to 280 μL of the DPPH solution. The mixture was incubated for 30 min and read at 515 nm using a microplate.

The 2,2′-azino-bis (3-ethylbenzothiazoline-6-sulfonic acid) (ABTS^•+^) radical cation was generated by reacting 5 mL of ABTS reagent with 88 μL of 140 mM potassium persulfate (K_2_S_2_O_8_). This mixture was incubated in the dark for 16 h to allow radical development. Subsequently, the stock solution was diluted with distilled water to obtain a working solution with an absorbance of 0.70 ± 0.02 at 734 nm. For the assay, 10 μL of the methanolic extract were combines with 290 μL of the adjusted ABTS^•+^ working solution. After a 6 min reaction period, the absorbance was measured at 734 nm.

A standard curve of ascorbic acid was used for both assays. The results for DPPH and ABTS were reported as mg of ascorbic acid equivalents (AAE) per gram of dry basis.

### 2.6. Moisture Sorption Isotherm Determination

Moisture sorption isotherms were determined using a Vapor Sorption Analyzer (Decagon Devices, Pullman, WA, USA) operating under the Dynamic Dewpoint Isotherm (DDI) mode. Approximately 1500 mg of sample were evenly spread in a stainless–steel sample pan to ensure uniform mass transfer and minimize internal diffusion resistance. The isotherms were performed at 25 °C, and water activity (a_w_) was varied over a range of 0.1 to 0.92 following an active adsorption–desorption loop sequence. The water activity step resolution was set at 0.01 a_w_. The initial moisture content of each sample was determined by the oven drying method at 105 °C until constant weight, in accordance with the AOAC 925.10 method for solids and flours.

The obtained sorption data from both adsorption and desorption runs were later fitted to two widely used mathematical models, namely Guggenheim–Anderson–de Boer (GAB, Equations (6) and (7), where M and M_0_ are equilibrium and monolayer moisture content; C and K are parameters related to heat sorption in the monolayer and multilayer region, respectively) and Peleg equations, to describe the relationship between equilibrium moisture content and a_w_ [24]. Model parameters were estimated using the Levenberg–Marquardt (LM) optimization implemented in the nlsLM function of the minpack.lm package in R [27]. The goodness of fit for each model was evaluated based on the mean squared error (MSE, Equation (8), where n is the number of datapoints, $M_{exp,i}$ and $M_{pred,i}$ are the experimental and predicted moisture content at point i, respectively) and the coefficients determination (R^2^, Equation (9), where *y_i_* and $\hat{y}$_i_ are the moisture experimental observation and estimated value, respectively).

(6) $$M=\frac{M_{0}CKa_{w}}{\left(1-Ka_{w})(1+(C-1)Ka_{w}\right)}$$

(7) $$M=k_{1}{a_{w}}^{n_{1}}+k_{2}{a_{w}}^{n_{2}}$$

(8) $$MSE=\frac{1}{n}\sum_{i=1}^{n}{\left(M_{exp,i}-M_{pred,i}\right)}^{2}$$

(9) $$R^{2}=1-\frac{\sum {\left(y_{i}-{\hat{y}}_{i}\right)}^{2}}{\sum {\left(y_{i}-\bar{y_{i}}\right)}^{2}}$$

### 2.7. Statistical Analysis

The study was conducted using a single-factor completely randomized design (CRD), where the independent variable was the source of the by-product (pulp, peel, or seed). All analytical determinations were performed in triplicate (n=3), and the results are expressed as mean values ± standard deviation (SD).

Prior to analysis, the data were tested for normality using the Anderson-Darling test. Data analysis was performed using one-way Analysis of Variance (ANOVA) to determine significant differences among the fractions. When significant differences were detected, means were compared using Tukey’s post hoc test with a significance level of α=0.05. Additionally, Pearson’s correlation coefficients (*r*) were calculated to evaluate the strength and direction of the linear relationships between the fiber composition and the techno-functional or antioxidant properties.

Furthermore, a Principal Component Analysis (PCA) was conducted to explore multivariate relationships among compositional, functional, and antioxidant variables. Prior to PCA, all variables were auto scaled (mean-centered and standardized to unit variance), and the analysis was performed using the correlation matrix.

All statistical analyses were performed using Minitab^®^ Statistical Software (Version 19, Minitab Inc., State College, PA, USA).

## 3. Results and Discussion

### 3.1. Physicochemical Composition

The values for physical dimensions, moisture, TA, pH, and soluble solids measurements are shown in Table 1. These parameters were used to determine the ripening stage of the guamuchil pods. The length (4.84 ± 1.04 cm) and width (5.31 ± 50 cm) differed from those reported in the literature, which ranged from 10 to 23 cm and 1.5 cm, respectively [3,28,29]. However, these differences may be attributed to the location where the data was collected, the maturity of the pods, and the manner in which the pods were analyzed. The pH value was 4.88 ± 0.02, which is similar to the value reported by Pandya and Mehta [28] of 4.86 ± 0.003. The values for TA and soluble solids were 2.26 ± 0.20 and 7.65 ± 0.06, respectively. The maturity index was (3.38 ± 0.14) and it has not been reported earlier [3,28]. The moisture content of the pod fractions ranged from 30.59 ± 3.78 g 100 g^−1^ db (GuPe) to 71.97 ± 1.71 g 100 g^−1^ db (GuP). The value for GuP conformed to that reported in the literature (70.3–77.8%); however, the report for GuS (10.7%) differed significantly and no values were detected for peel moisture [3,28,30].

### 3.2. DFC Characterization

#### 3.2.1. Dietary Fiber Composition of DFC

The dietary fiber composition is presented in Table 2. Every dietary fiber fraction showed significant differences (*p* < 0.05) among the DFC, except for the high- and low-molecular-weight dietary fibers. The dietary fiber concentrate with the highest content of TDF was GuPe with 64.16 ± 1.23 g 100 g^−1^ db, followed by GuS and GuP. The IDF content presented similar behavior to that of the TDF. On the contrary, for SDF, the sequential order was GuS > GuPe > GuP, where the value for GuS was 9.42 ± 0.32 g 100 g^−1^ db. For SDFP and SDFS, important differences were found. No significant differences (*p* < 0.05) were noted in the amount of SDFP in GuPe and GuS, albeit the quantity of SDFS was significantly higher in GuS, while GuP and GuPe did not present any significant differences. This finding can be attributed to the important oligosaccharide fractions detected in the legume seeds such as raffinose and stachyose [31].

The SDF:IDF and SDF:TDF ratios are important parameters used to determine the composition of food ingredients. Previously, similar values to ours have been reported for SDF:TDF ratio for different fruits and legumes, such as banana, mamey sapote, orange, prickly pear, and tamarind, ranging from 0.06 to 0.29 [18]. Soluble dietary fiber has demonstrated greater acceptance for texture, flavor, and greater hydration properties when incorporated into foods and drinks [17]. Knowing the exact content of the fiber in this legume for each fraction allows the selection of functionality for the powder as an ingredient with the ability to modify the overall texture and the rheological and sensory qualities [32].

#### 3.2.2. Proximate Characterization of DFC

The proximate characterization of guamuchil DFC is shown in Table 3. All parameters were reported on a dry basis. The moisture content ranged from 1.12 ± 0.39 (GuP) to 4.87 ± 1.04 (GuP) g 100 g^−1^ db with no significant differences (*p* < 0.05) between the GuP and GuS fractions. Rao et al. [33] reported values almost 3.5-times greater for GuP moisture (14.7–17.8%); nevertheless, Flores-Jiménez et al. [10] reported similar values for the GuS fraction; these differences in the results can be attributed to the different drying methodologies used. The highest lipid content was detected in the GuS (13.80 ± 0.91 g 100 g^−1^ db) fraction. The seeds are known to have high-fat content and have been reported as a novel source of edible oil owing to their 9 saturated and 17 unsaturated fatty acids [29,34]. The ash content ranged from 2.03 ± 0.18 g 100 g^−1^ db (GuP) to 5.67 ± 0.22 (GuPe) g 100 g^−1^ db, which is similar to the values reported by Rao et al. [33] and Roselin and Parameshwari [29], ranging from 2.4% to 5%.

The maximum protein content was exhibited by GuS, with a value of 19.53 ± 0.42 g 100 g^−1^ db, which is highly similar to the protein content (22.2%) reported by Narsing-Rao et al. [12] for the same fraction. The GuP fraction presented 12.47 ± 0.30 g 100 g^−1^ db in protein content, which conforms to the values reported in the literature of 12–15% [6,33]. Finally, the protein content in GuPe was 11.96 ± 0.53 g 100 g^−1^ db, which could not be compared owing to the lack of comparative data. DFC from the GuP fraction showed the highest content of digestible carbohydrates (67.10 ± 1.12 g 100 g^−1^ db), followed by GuS and GuPe (20.91 ± 1.18 and 16.23 ± 0.71 g 100 g^−1^ db, respectively); these values are in accordance with those reported by Garcia-Amezquita et al. [18] for DFC obtained from different fruits and legumes pulp and their byproducts.

#### 3.2.3. Water Activity (a_w_) and Color of DFC

Water activity is an important parameter for the product’s shelf life; this parameter is highly related to product deterioration by enzymes, microorganisms, lipid oxidation, and nonenzymatic reactions. This parameter describes the available or free water in the food products [35]. The a_w_ differs significantly (*p* < 0.05) among the DFC fractions (Table 2); nevertheless, the values were determined as >0.42, indicating that these DFCs are not at risk of developing pathogenic microorganisms or other reactions that can increase their deterioration [36]. Lightness (L) and the chromatic coordinates (a b) for DFC obtained from the guamuchil fractions are depicted in Table 2, where minor significant differences (*p* < 0.05) were observed among the fractions’ luminosity. In contrast, large significant differences were found in the values for a* (red color) and b* (yellow color) among the DFC powders, as well as the same relationship order of GuP > GuPe > GuS.

### 3.3. Techno-Functional Characterization of DFC

**Figure 2:**
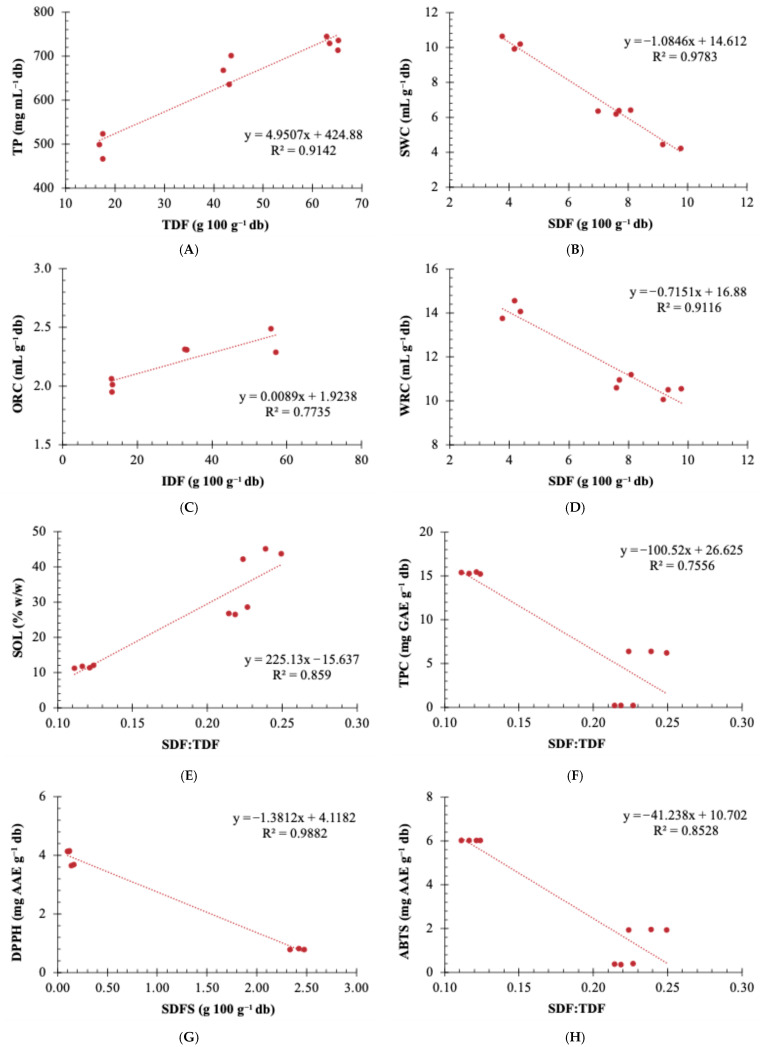
Correlation among functional properties and different dietary fiber fractions: (**A**) Tapping density (TD) and TDF, (**B**) Swelling capacity (SWC) and SDF, (**C**) Oil retention capacity (ORC) and IDF, (**D**) Water retention capacity (WRC) and SDF, (**E**) Solubility (SOL) and SDF:TDF, (**F**) Total phenolic content (TPC) and SDF:TDF, (**G**) DPPH and SDFS, and (**H**) ABTS and SDF:TDF of Guamuchil fractions DFC (GuP, GuPe, and GuS). TDF: total dietary fiber, IDF: insoluble dietary fiber, SDF: soluble dietary fiber, SDFS: low molecular weight soluble dietary fiber, GAE: gallic acid equivalents, AAE: ascorbic acid equivalents.

The techno-functional properties of the Guamuchil DFCs (TD, WRC, ORC, SWC, and SOL) are summarized in Table 4, while the Pearson correlations between properties and the fiber composition are illustrated in Figure 2.

TD reflects the properties such as flowability and cohesion in flours and powders that are useful for packaging purposes in the industry. Significant differences (*p* < 0.05) were observed among fractions, with the peel DFC (GuPe) exhibiting the highest density (730 ± 13 mg cm^−3^ db) followed by the seed (GuS) and the pulp (GuP). Similar results were observed for fava beans, chickpeas, and lentils, as well as for fruits like oranges, mango, and prickly pear [23,37,38]. A strong positive correlation (R^2^ = 0.91) was found between TDF content and TD (Figure 2A). This indicates that the rigid, fibrous matrix of the peel and seed allows for a more compact arrangement compared to the pulp, which likely contains amorphous sugars that create irregular voids. The TD value of GuPe is superior to those reported for other Latin American fiber sources, such as mango peel [16] and prickly pear peel [39], suggesting a competitive advantage for GuPe in terms of storage efficiency and transport cost reduction for the food industry.

Regarding SWC, the order was GuP > GuPe > GuS, with values ranging from 10.23 to 4.35 mL g^−1^ db, respectively. López-Marcos et al. [40] reported lower values for different fiber-rich agro-industrial coproducts such as pomegranate (1.92 mL g^−1^ db) and tiger nut (1.93 mL g^−1^ db. Similarly, the SWC value reported for *Tamarindus indica* (2.7 mL g^−1^ db) is 3.8-times smaller than the GuP value; additionally, *T. indica* has been evaluated for bakery purposes with positive outcomes, meaning that DFC from Guamuchil has the potential for use in food industry [41,42].

An important negative correlation (R^2^ = 0.99) was detected between SWC and SDF composition (Figure 2B). This phenomenon can be attributed to the specific profile of the soluble fraction in the GuS. As shown in Table 2, this fraction possesses the highest SDF and SDFS content. The SDFS components, likely consisting of non-digestible oligosaccharides typical of legume seeds (e.g., raffinose/stachyose), possess high water solubility but lack the polymeric chain length required to form three-dimensional networks that entrap water and expand the matrix volume. Consequently, these molecules dissolve into the supernatant rather than contributing to the sediment volume measured as SWC. In contrast, the pulp (GuP), despite having lower total SDF, exhibited the highest SWC, suggesting its soluble fraction comprises SDFP, likely pectins), capable of forming viscous, water-holding gels [43,44].

ORC is another characteristic of high-fiber ingredients that is important for food formulations; this property presents beneficial effects on stabilizing high-fat products and emulsions. ORC followed an inverse trend, being significantly higher (*p* < 0.05) in the peel and seed (2.31 mL g^−1^ db) fractions compared to the pulp (1.98 mL g^−1^ db). Several studies have reported similar values for ORC using different high-fiber powders obtained from diverse fruit and vegetable fractions [23,39,40,45]. Moreover, a positive correlation (R^2^ = 0.71) was noted between IDF and ORC (Figure 2C), as also reported by Tejada-Ortigoza et al. [46] and López-Marcos et al. [40]. This mechanism is attributed to the hydrophobic nature of insoluble fibers (lignin/cellulose) and their porous surface structure, which physically entrap oil droplets [17,40]. This functional attribute positions Guamuchil peel and seed DFCs as suitable ingredients for stabilizing high-fat matrices, such as meat analogs, sausages, or emulsified dressings, where they can improve mouthfeel and prevent fat separation during cooking [47].

Finally, the interplay between WRC and SOL further elucidated the impact of fiber structure. The studied DFC revealed a good capacity to bind water, with WRC values ranging from 10.32 ± 0.24 to 14.17 ± 0.35 mL g^−1^ db for GuS and GuP, respectively. These values are superior to those reported in previous studies, such as DFC obtained from orange, mango, and prickly pear (up to 4.4 mL g^−1^ db) [23], and micronized grape pomace fibers (up to 2.56 mL g^−1^ db) [48]. A negative correlation (R^2^ = 0.91, Figure 2D) was noted between WRC and SDF. This behavior parallels the trend observed in swelling capacity and can be attributed to the molecular nature of the soluble fractions: in the seed, the abundant SDFS dissolve completely into the liquid phase during the WRC assay centrifugation, reducing the water retained in the insoluble pellet [23,40].

This is corroborated by the solubility index results, where GuP displayed the highest functionality (43.54 ± 1.22%), coinciding with the highest SDF:TDF ratio. A positive correlation was identified between SOL and the SDF:TDF ratio (R^2^ = 0.86, Figure 2E), confirming that a higher proportion of soluble fiber enhances dispersibility. The SOL values for GuS align with those reported for legume flours like lentil (20.17 to 24.8%) [49], while the high solubility of the GuP fraction is comparable to soluble- rich fibers from mango, orange, and prickly pear peels (55.1% to 83.9%) [39], supporting its potential application in beverages and liquid formulations.

### 3.4. Bioactivity of DFC

The total phenolic content (TPC) and antioxidant activity (assessed via DPPH and ABTS assays) of the Guamuchil fractions are presented in Table 4. The TPC values exhibited a significant decreasing order (*p* < 0.05): GuPe (15.46 ± 0.26 mg GAE g^−1^ db) > GuP (6.25 ± 0.11 mg GAE g^−1^ db) > GuS (0.23 ± 0.02 mg GAE g^−1^ db). Notably, the TPC of the GuPe is remarkably higher than values reported for other fruit by-products commonly valorized as functional ingredients, such as grape pomace (3–7.5 mg GAE g^−1^) and mango peel (approx. 11.2 mg GAE g^−1^) [19,45,48]. Furthermore, the pulp (GuP) values in this study align with the range of 6.3–11.5 mg GAE g^−1^ reported for *Pithecellobium dulce* arils processed under different drying methods [50]. This indicates that both the peel and pulp of Guamuchil are competitive sources of phenolic compounds within the Latin American biodiversity context, comparable to recognized superfruits like berries or pomegranate [51].

Regarding antioxidant activity, the peel fraction consistently displayed the highest scavenging capacity for both DPPH (4.13 ± 0.03 mg AAE g^−1^ db) and ABTS (6.01 ± 0.01 mg AAE g^−1^ db), followed by the pulp and the seed, showing a strong positive correlation with TPC, indicating that the increase in the antioxidant capacity is powered by the TPC, as has also been reported by previous studies using different types of samples [20,21,45].

To elucidate the interaction between the fiber matrix and bioactivity, Pearson correlations were analyzed (Figure 2F–H). Negative correlations were found between the SDF:TDF ratio and both TPC and ABTS. This behavior indicates that higher antioxidant potential is associated with fractions possessing a lower proportion of soluble fiber (and consequently, a higher proportion of IDF), such as the peel. This finding aligns with the biological function of fruit peels, which develop a dense lignocellulosic matrix (rich in IDF) accumulated with high concentrations of extractable phenolics —such as flavonoids and hydrolysable tannins—to act as a defense barrier against environmental stress [52].

Conversely, the seed DFC (GuS) presented the lowest bioactivity. This fraction is characterized by the highest content of SDFS (2.41 ± 0.07 g 100 g^−1^ db), likely composed of non-digestible oligosaccharides typical of legume seeds (e.g., raffinose and stachyose). A negative correlation was observed between SDFS and DPPH (Figure 2G). This suggests that these specific soluble carbohydrates do not contribute to the antioxidant capacity; rather, their high concentration in the seed dilutes the overall bioactivity. Furthermore, the phenolic compounds present in the seed are likely insoluble-bound phenolics covalently linked to the cell wall, rendering them resistant to the standard methanolic extraction used in this study [53]. Recent evidence confirms that while legume seeds contain significant phenolics, they require hydrolysis to be released and quantified [54].

Consequently, the Guamuchil peel DFC stands out as the superior functional ingredient, where the bioaccessible antioxidants are readily available and not hindered by the high oligosaccharide content or strong covalent bonding observed in the seed.

### 3.5. Multivariate Analysis of Physicochemical and Techno-Functional Profiles of Guamuchil Fractions

**Figure 3:**
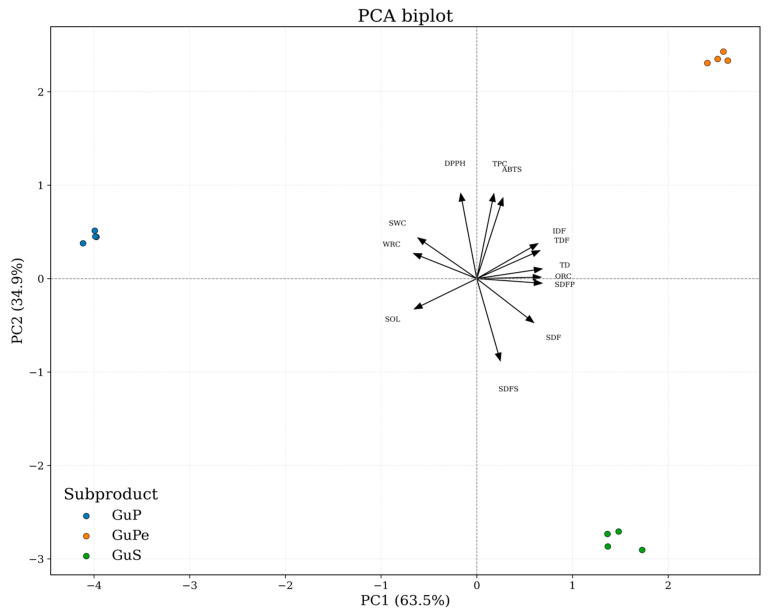
Principal Component Analysis (PCA) biplot of the relationships among the physicochemical and functional properties of Guamuchil fractions— pulp (GuP), peel (GuPe), and seed (GuS). Samples are plotted according to their scores on PC1 and PC2, while vectors represent the loadings of each variable: tapping density (TD), swelling capacity (SWC), oil retention capacity (ORC), water retentions capacity (WRC), solubility (SOL), total phenolic compounds (TPC), DPPH (2,2-diphenyl-1-picryl-hydrazyl-hydrate, ABTS: (2,2′-azino-bis (3-ethylbenzothiazoline-6-sulfonic acid)).

The multivariate relationships among the physicochemical, techno-functional, and antioxidant variables of the guamuchil fractions are summarized in Figure 3. The PCA clearly clustered the pulp (GuP), peel (GuPe), and seed (GuS) fractions along the first two principal components, which explained 63.5% (PC1) and 24.9% (PC2) of the total variance. Samples from the pulp fraction grouped on the negative side of the PC1 and aligned strongly with the vectors of solubility (SOL), swelling capacity (SWC), and water retention capacity (WRC), reflecting the influence of soluble fiber and amorphous polysaccharides [17,55]. In contrast, the peel (GuPe) grouped with total phenolic content and antioxidant activity (DPPH, ABTS), which is consistent with the high phenolic accumulation typically observed in legume peels [56,57]. The seed fraction (GuS) occupied an intermediate position, indicating a balanced contribution from structural carbohydrates and moderate hydration properties [58]. Overall, the PCA highlights the compositional drivers that differentiate the functional potential of each fraction, supporting their targeted use in food applications.

### 3.6. Moisture Sorption Isotherms of DFC

**Figure 4:**
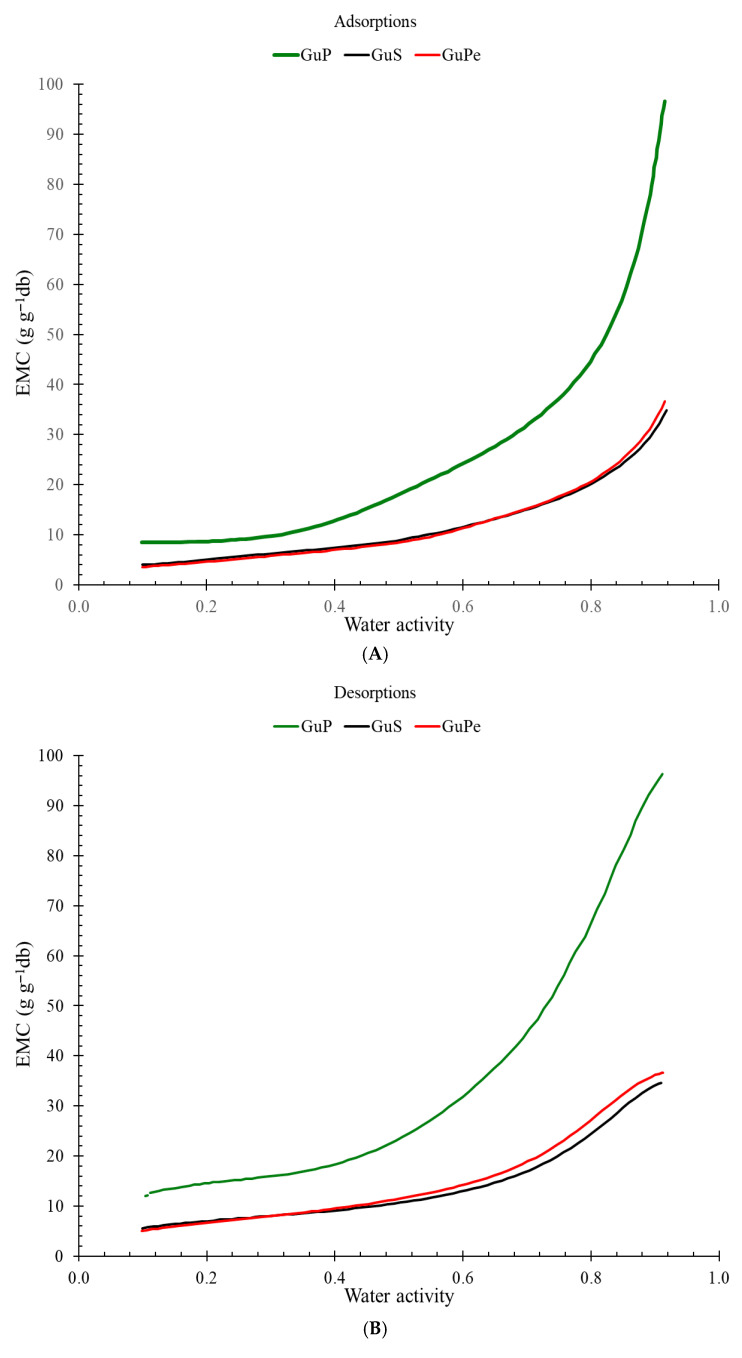
Moisture (**A**) adsorption and (**B**) desorption isotherm at 25 °C from *P. dulce* fractions. Equilibrium moisture content (EMC) of Guamuchil pulp (GuP), peel (GuPe), and seed (GuS).

The equilibrium moisture content (EMC) of the guamuchil fractions as a function of water activity at 25 °C is presented in Figure 4 for both adsorption (a) and desorption (b) processes. All samples exhibited type II sigmoidal isotherms according to the Brunauer classification, which is characteristic of food matrices rich in carbohydrates and proteins. At low water activity (a_w_ < 0.3), a gradual increase in EMC was observed, corresponding to the monolayer region where water molecules are strongly adsorber onto polar sites of biopolymers [59]. As a_w_ increase above 0.6, a sharp rise in EMC occurred, indicating the multilayer and capillary condensation regions associated with plasticization of amorphous components and the onset of structural relaxation phenomena [59,60].

Among the evaluated fractions, GuP exhibited the highest EMC values across the entire a_w_ range during both adsorption and desorption, suggesting a greater availability of hydrophilic constituents and amorphous carbohydrates capable of retaining water. This trend is consistent with its previously reported techno-functional properties related to water, such as WRC and SWC, in which GuP significantly outperforms the other two fractions. This behavior can be attributed to its higher content of soluble sugar and polysaccharide content. Conversely, GuS and GuPe displayed similar behavior, with lower hygroscopicity than GuP, likely due to the higher and polyphenolic content in GuPe and the higher fat and protein content in GuS [22,61].

The experimental data were successfully fitted to both the GAB and Peleg models, with model performance and parameters summarized in Table 5. The goodness of fit indicators (MSE = 0.058–1.303 and R^2^ ≥ 0.997 for adsorption; MSE = 0.708–11.660 and R^2^ ≥ 0.999 for desorption) demonstrated that both models adequately described the sorption behavior of all fractions. The GAB model provided slightly lower MSE values for adsorption, while the Peleg model better captured the desorption phase.

The monolayer moisture content (M_0_) estimated using the GAB equation ranged from 5.38 to 17.27 g H_2_O g^−1^ db, indicating the amount of water strongly bound to the most active sorption sites of the matrix [59]. Higher M_0_ values in GuP suggest a greater density of active polar sites, consistent with its higher content of soluble and amorphous carbohydrates capable of hydrogen bonding [61]. Similar trends have been reported in other fruit pulps rich in polysaccharides and low-molecular-weight sugars, which promote hygroscopicity via hydrogen bonding [62,63]. Conversely, GuPe and GuS exhibited lower M_0_ values, reflecting fewer accessible sorption sites due to their denser structures [64]. The C parameter, associated with sorption heat and the strength of water–solid interactions [65], was relatively high for GuS and GuPe (14.18–26.11), suggesting that at low water activity, these matrices retain a higher proportion of strongly bound water, likely due to rigid structural components such as lignin, proteins, and condensed polyphenols [22]. Comparable patterns have been reported in high-fiber or phenolic-rich residues, where lower total water uptake is accompanied by stronger initial binding energies [66].

The Peleg model, which offers an empirical but flexible description across the full sorption range, also showed excellent agreement with the experimental data (R^2^ ≥ 0.997 for all cases), in line with previous studies on complex plant matrices [61,62,67,68]. Higher k_1_ coefficients during adsorption, particularly for GuP (171.32), indicate greater moisture uptake, whereas lower n_1_ and n_2_ values for GuPe and GuS reflect a less pronounced moisture increase at intermediate a_w_, consistent with their denser composition [62].

Overall, the modeling results confirm that the water sorption behavior of guamuchil fractions is strongly governed by their chemical composition and matrix structure. The GuP fraction, due to its greater hygroscopicity, is suitable for applications requiring high water–binding capacity or hydration (e.g., bulking or texturizing agents). Meanwhile, GuPe and GuS, which exhibited lower moisture retention, are more appropriate for formulations demanding higher moisture stability and structural rigidity [22,66].

## 4. Conclusions

This study provides the first comprehensive characterization of *Pithecellobium dulce* (guamuchil) fractions—pulp, peel, and seed—demonstrating their viability as novel sources of dietary fiber concentrates (DFC) with distinct techno-functional profiles. The physicochemical analysis revealed that the specific fiber composition governs the functionality of each fraction, establishing a clear structure–function relationship essential for industrial upscaling.

The pulp DFC (GuP) emerged as a superior hydrocolloid-type ingredient. Its high SDF content resulted in exceptional hydration properties, reaching a solubility of 43.54% and a WRC of 14.17 mL g^−1^ db, values that are comparable to or exceed those of established commercial hydrocolloids like *Opuntia* mucilage. This quantitative profile suggests that GuP has significant potential as a natural texturizer, viscosity enhancer, or syneresis inhibitor in bakery and dairy formulations, aligning with the growing consumer demand for “clean label” additives.

The peel DFC (GuPe) was identified as a high-value antioxidant fiber, its total phenolic content (15.46 mg GAE g^−1^ db) and antioxidant capacity surpass those of common fruit by-products such as grape pomace. This fraction represents a strategic ingredient for the functional foods market, offering a dual benefit: dietary fiber enrichment and stability improvement. Furthermore, the seed DFC (GuS), despite showing lower antioxidant extractability due to bound phenolics, presented the highest protein content (∼19.5%) and a competitive ORC (2.31 mL g^−1^ db). This profile positions it as a promising candidate for the development of plant-based meat analogs or emulsified systems, where structural integrity and fat retention are critical.

The moisture sorption isotherms demonstrated that while the pulp is highly hygroscopic (monolayer moisture M_0_ > 10 g 100 g^−1^ db), the peel and seed fractions exhibit superior stability at high water activities (M_0_ < 7.8 g 100 g^−1^ db), a crucial factor for industrial storage and transport logistics. Future research should prioritize the evaluation of the in vitro bioaccessibility of the bound phenolics in the seed, toxicological safety assessments, and the validation of these ingredients in real food systems to consolidate the transition of Guamuchil from an underutilized crop to a high-value industrial resource.

## Figures and Tables

**Table 1:** Physicochemical composition of Guamuchil (*Pithecellobium dulce*) pod and fractions.

Parameter	Units	Measurement
Physical dimensions		
Length	cm	4.84 ± 1.04
Width	cm	5.31 ± 0.50
Weight	g	18.73 ± 5.16
pH		4.88 ± 0.02
Titratable acidity (TA)	g CA 100 g^−1^ wb	2.26 ± 0.20
Soluble solids (SS)	°Brix	7.65 ± 0.06
Maturity index (SS TA^−1^) *		3.38 ± 0.14
Moisture		
Pulp	g 100 g^−1^ wb	71.97 ± 1.71
Peel	g 100 g^−1^ wb	30.59 ± 3.78
Seed	g 100 g^−1^ wb	44.83 ± 6.78

* Base in the method reported by Garcia-Amezquita et al. [28]. Values are the means of at least 4 independent experiments ± standard deviation. CA: citric acid, wb: wet basis.

**Table 2:** Dietary fiber composition of Guamuchil (*Pithecellobium dulce*) DFC.

Pod Fraction	TDF g 100 g^−1^ (db)	IDF	SDF	SDFP	SDFS	SDF:TDF
Pulp (GuP)	17.29 ± 0.39 ^c^	13.18 ± 0.12 ^c^	4.11 ± 0.31 ^c^	4.01 ± 0.38 ^b^	0.15 ± 0.02 ^b^	0.24:1
Peel (GuPe)	64.16 ± 1.23 ^a^	56.57 ± 0.94 ^a^	7.59 ± 0.45 ^b^	7.51 ± 0.40 ^a^	0.11 ± 0.01 ^b^	0.12:1
Seed (GuS)	42.84 ± 0.84 ^b^	33.42 ± 0.74 ^b^	9.42 ± 0.32 ^a^	7.01 ± 0.25 ^a^	2.41 ± 0.07 ^a^	0.22:1

Values are the means of three independent experiments ± standard deviation. TDF: Total dietary fiber, IDF: Insoluble dietary fiber, SDF: Soluble dietary fiber, SDFP: High molecular weight soluble dietary fiber, SDFS: Low molecular weight soluble dietary fiber, db: dry basis. Dietary fiber composition is expressed in g 100 g^−1^ db. Different letters within the same column are significantly different (*p* < 0.05) according to Tukey’s Test.

**Table 3:** Physicochemical and proximate characterization of DFC obtained from Guamuchil pod fractions.

Parameter	Units	Pulp (GuP)	Peel (GuPe)	Seed (GuS)
Moisture	g 100 g^−1^ db	4.87 ± 1.04 ^a^	1.12 ± 0.39 ^b^	4.75 ± 0.56 ^a^
Protein	g 100 g^−1^ db	12.47 ± 0.30 ^b^	11.96 ± 0.53 ^b^	19.53 ± 0.42 ^a^
Ash	g 100 g^−1^ db	1.95 ± 0.17 ^c^	5.48 ± 0.20 ^a^	2.90 ± 0.14 ^b^
Fat	g 100 g^−1^ db	1.56 ± 0.14 ^c^	2.65 ± 0.07 ^b^	13.80 ± 0.91 ^a^
TDF	g 100 g^−1^ db	17.29 ± 0.39 ^c^	64.16 ± 1.23 ^a^	42.84 ± 0.84 ^b^
Digestible carbohydrates	g 100 g^−1^ db	67.10 ± 1.12 ^a^	16.23 ± 0.71 ^c^	20.91 ± 1.18 ^b^
a_w_		0.41 ± 0.00 ^b^	0.42 ± 0.02 ^a^	0.40 ± 0.01 ^c^
Color				
L		39.70 ± 0.29 ^c^	42.54 ± 0.08 ^a^	40.34 ± 0.30 ^b^
a		6.61 ± 0.06 ^a^	2.42 ± 0.08 ^b^	1.76 ± 0.06 ^c^
b		19.27 ± 0.05 ^a^	15.56 ± 0.13 ^b^	8.29 ± 0.11 ^c^

Data are the means of three independent experiments ± standard deviation. db: dry basis. Different following letters within the same row are significantly different (*p* < 0.05) according to Tukey’s Test.

**Table 4:** Functional properties of guamuchil (*Pithecellobium dulce*) pod fractions.

Parameter	Units	Pulp (GuP)	Peel (GuPe)	Seed (GuS)
TD	mg mL^−1^	495.45 ± 22.92 ^c^	730.11 ± 13.40 ^a^	667.92 ± 26.66 ^b^
SWC	mL g^−1^	10.23 ± 0.30 ^a^	6.33 ± 0.10 ^b^	4.35 ± 0.13 ^c^
ORC	mL g^−1^	1.98 ± 0.08 ^b^	2.31 ± 0.16 ^a^	2.31 ± 0.01 ^a^
WRC	mL g^−1^	14.17 ± 0.35 ^a^	10.73 ± 0.43 ^b^	10.32 ± 0.24 ^b^
SOL	% (w w^−1^)	43.54 ± 1.22 ^a^	11.57 ± 0.42 ^c^	26.97 ± 1.09 ^b^
TPC	mg GAE g^−1^	6.25 ± 0.11 ^b^	15.46 ± 0.26 ^a^	0.23 ± 0.02 ^c^
DPPH	mg AAE g^−1^	3.62 ± 0.07 ^b^	4.13 ± 0.03 ^a^	0.80 ± 0.03 ^c^
ABTS	mg AAE g^−1^	1.95 ± 0.03 ^b^	6.01 ± 0.01 ^a^	0.40 ± 0.04 ^c^

Values are the means of four independent experiments ± standard deviation. TD: Tapping density, SWC: Swelling capacity, ORC: Oil retention capacity, WRC: Water retention capacity, SOL: Solubility. TPC: Total Phenolic compounds, DPPH: 2,2-diphenyl-1-picryl-hydrazyl-hydrate, ABTS: 2,2′-azino-bis (3-ethylbenzothiazoline-6-sulfonic acid). GAE: gallic acid equivalents, AAE: ascorbic acid equivalents. Results are expressed in dry basis. Different letters within the same row are significantly different (*p* < 0.05) according to Tukey’s Test.

**Table 5:** Coefficients and statistical evaluation of GAB and Peleg models for adsorption and desorption moisture sorption isotherms of *P. dulce* fractions.

Model		Pulp (GuP)		Peel (GuPe)		Seed (GuS)	
		Adsorption	Desorption	Adsorption	Desorption	Adsorption	Desorption
GAB	M_0_	10.258	17.272	5.382	7.761	5.439	6.521
	C	7.661	7.330	9.673	9.323	14.185	26.112
	K	0.977	0.914	0.933	0.884	0.920	0.902
	MSE	1.303	11.660	0.165	1.025	0.058	0.708
	R^2^	0.998	0.985	0.997	0.990	0.999	0.991
Peleg	k1	171.317	116.472	12.584	39.393	12.882	38.009
	n1	12.797	3.841	0.621	3.985	0.578	4.474
	k2	45.007	16.700	39.118	11.078	35.274	10.673
	n2	1.232	0.117	6.038	0.320	6.035	0.264
	MSE	2.249	0.752	0.231	0.249	0.165	0.153
	R^2^	0.997	0.999	0.997	0.998	0.997	0.998

MSE: mean squared error; R^2^: Pearson’s coefficient of determination.

## Data Availability

The original contributions presented in the study are included in the article. Further inquiries can be directed to the corresponding author.

## Abbreviations

The following abbreviations are used in this manuscript:

GuP	Guamuchil Pulp
GuPe	Guamuchil Peel
GuS	Guamuchil Seed
IDF	Insoluble Dietary Fiber
ORC	Oil retention capacity
SDF	Soluble Dietary Fiber
SDFP	Soluble Dietary Fiber high molecular weight fraction
SDFS	Soluble Dietary Fiber low molecular weight fraction
SOL	Solubility
SWC	Swelling capacity
TD	Tapping density
TDF	Total Dietary Fiber
TPC	Total phenolic compounds
WRC	Water retention capacity
